# Medical Miscellanies

**Published:** 1816-12

**Authors:** 


### PART IV.

### MEDICAL MISCELLANIES.

Quicquid agunt Homines.

### MORBID ANATOMY.

(Selected and abridged from MORGAGNI.)

WITH COMMENTARIES.

(Continued from page 437.)

"Wherever Morbid Anatomy has been steadily adhered to as the foundation of observation and reasoning in our science, that science has been progressive: it has been interrupted in its course, even amidst the most brilliant reputation of its professors, wherever men have arisen, whose inventive powers have led them to reject, rather than to seek the aid of Anatomy."—*C. Bell, on the Diseases of the Urethra.*

### *Letter V. On Apoplexy arising from neither a sanguineous nor serous Cause.*

When Morgagni divided apoplexies into sanguineous and serous, he did not mean to divide the disease itself, "but rather the more frequent dissections of apoplectic bodies which had happened to others and himself," at the same time ingenuously confessing that other causes of apoplexies besides blood and serum were frequently found, and which it will be the object of this letter to shew.

CASE 1. A man, 33 years of age, of a sanguineous temperament, and much addicted to wine and tobacco, began to have a pain in the *left* side of his head, especially towards the occiput, which was followed by a pain and weakness in the muscles of the neck on the same side. Next came on a violent fever, which afterwards seemed to remit, his pulse becoming slow, weak, and easily compressible. His strength now decayed, and every motion of the body was performed with difficulty. After an interrupted delirium, aphonia succeeded, with inability to move; and in this state he lingered out till the fourteenth day, when he died.

*Sectio Cadaveris.* When the brain was taken out of the cranium, a little purulent matter was observed on its basis, and continued to ooze out there: in short, both the ventricles, but especially the *right*, overflowed with it,

In the corpus striatum of this ventricle was a foramen which communicated with a sinous ulcer that occupied a third part of the basis of the brain on the right side. The left side of the brain was sound.

Remarks. Modern Pathologists will not be apt to call this a case of apoplexy at all; but simply an inflammation in the brain, terminating in abscess.

Case 2. A woman, above 40, after having the catamenia greatly diminished, was attacked with a cancerous tumour in her left leg; which ulcerating, considerable pain, and almost continual fever came on. She had suffered thus a year and more, when, quite worn out with her sufferings, she begged Valsalva to amputate the leg, which he did.* On the third day the stump appeared livid; but, by the application of stimulants, a lively colour was restored to the parts in two days. Meanwhile, the fever became, every day, more violent and acute. The right parotid gland swelled and inflamed so much, that for twenty-four hours she could not swallow even liquids. At length, profuse perspirations coming on, the fever remitted; the parotid swelling was discussed, and every thing seemed to proceed well. About the thirtieth day, however, in consequence of some irregularity in diet, the acute fever returned, lasted some days, and was again carried off by perspiration. All this time the cure of the wound went on well, till at length the cicatrix was all but closed. About the third month after amputation, when the patient was one day taken out of bed, she was seized with a kind of apoplexy. To a diminution of sensation and motion in the whole right side of the body, were added delirium and convulsions, which, after some remissions and exacerbations, carried off the patient in a few days.

*Sectio Cadaveris.* The skull being opened, a large quantity of pus was found stagnating in the left ventricle of the brain; but no trace of injury or disorganization could be discovered in any part of the brain itself!

Remarks. The explication of this phænomenon puzzles Morgagni very much; and no wonder, for till the present moment, no physiologist or pathologist can demonstrate *how* matter is taken up from one part and deposited in another; or how a surface or set

---

* It appears from this, that within a century, one of the greatest physicians of Italy united physic and surgery. Now that the two sciences are so *immeasurably* distinct, have we greater men than Valsalva?

of vessels which, during a long life, secreted nothing but lymph, will, all at once, change their function, and pour out pus! Yet, such is the case; and whether we call it *metastasis* of matter, or metastasis of action, they are only abstract terms to designate a *fact*, and hide our own ignorance of the *cause*.

Morgagni, however, reasons *nearly* as well as a first rate modern pathologist. "It might be suspected (says he) that the matter which had previously been carried to the leg, was now carried into the brain." "But the matter which had been before carried into the leg, was made *pus* in the *ulcers* of the leg; whereas, in the brain, no ulcer was found." "I should rather suspect, that at the time of the acute fevers, matter was collected in some internal part of the body, and an abscess was formed."—In this last case he must mean, though he does not expressly say, that this matter was afterwards translated to the ventricle of the brain.

The case shews us, that the *total removal* of a part or limb to which an afflux of matter had long been established, is not a certain security against some future determination to some still more important organ; and consequently points out the propriety of substituting other drains. How often do we see these reasonings illustrated after the excision of cancers, &c. &c. or even the amputation of limbs.

Case 3. A woman of Padua, in the 59th year of her age, was seized with an apoplexy. To this, a violent fever succeeded, for which she was brought into the hospital. Although she could not speak, she understood on the first day; for she held out her *left* arm to the physicians, the right side being completely void of sensation or motion. Her face was red; no difficulty in swallowing. She died in a few days.

*Sectio Cadaveris.* The bladder, distended with urine, projected six inches above the pubis: it was inflamed, as was the meatus urinarius. The gall-bladder was turgid with brown bile, and contained some biliary concretions. All the other abdominal viscera natural. The right lobe of the lungs, on the posterior part, diseased.

The skull-cap being removed, a quantity of serum came forth; the dura mater was thickened; the vessels of the pia mater injected with blood. Among the convolutions of the brain, under the pia mater, was seen a transparent water; the plexus choroides contained vesicles turgid with water, one of them as large as a grape. The left thalamus nervi optici of a brown colour, and the surrounding medullary substance soft, and as it were, liquified, and mixed with a bloody fluid. All the other parts of the cerebrum were of a natural colour, but firmer than usual.

Remarks. Morgagni considers this "an apostem sui generis," produced by repletion. He observes, that when apoplectic persons drag on life a little longer than usual, a new accession of disorders is frequently brought on from retention of urine, as in this case. The retention of urine, on such occasions, escapes the notice of superficial observers, because a dribbling, from weakness of the sphincter vesicæ, commonly accompanies it. Hence the necessity of examining the region of the bladder, and of drawing off the water, to prevent the sufferings of the patient alone, if no other object were in view.

Case. 4. A tailor, a great inebriate, being taken speechless, died in two days. No history could be obtained.

*Sectio Cadaveris.* There was nothing very particular in the thoracic or abdominal cavities. On severing the head from the trunk, much serum escaped from the vertebral tube, and the same was found under the pia mater in great quantities. A white inodorous sanies extended all over the anterior lobes of the brain, and was inherent in the very substance of the pia mater, the brain itself being sound, but its vessels exceedingly turgid.

Remarks. Morgagni seems to think this sanies was *translated* from the lungs, or some other part; but we see no reason why it should not be the product of inflammation or congestion in the brain itself, though we do not deny the doctrine of translation or metastasis.

Case 5. An Æthiopian at Venice, ætat. 30, muscular, and in good health, "unless that in the last months, he had been subject *to a certain languor of his stomach*, joined with a slight sweating, which went off on taking food," while playing on the trumpet, fell backwards very gradually and expired the moment that he fell.

*Sectio Cadaveris,* twelve hours after death. The neck was blacker than the other parts of the body, as if from stagnating blood. Abdominal viscera sound, except the edge of the liver, which was almost livid. The cartilages of the ribs were beginning to ossify. Both cavities of the chest contained more water than usual, as did the pericardium, in which it was thick and turbid. On the surface of the heart the blood-vessels were very conspicuous. In the brain, water was found under the pia mater, and a little more than usual in the lateral ventricles. No turgidity in the vessels of the brain. The blood-vessels passing over the corpus callosum were distended with air, intermixed with a little serum.

Remarks. It is highly probable that chronic inflammation of the heart had been going on here, and that the cerebral phænomena were not the entire cause of death. A small quantity of water suddenly effused in the pericardium will often produce the most fatal effects.

Case 6. A fisherman of Venice, about 40, tall, and afflicted with a rupture, being liable to flatulent complaints in his belly, was seized suddenly, as he sat in his boat, and instantly died.

*Sectio Cadaveris.* Stomach and intestines distended with air; stomach somewhat red. The trunk of the gastro-epliploicæ distended with blood and air, to the size of the fore finger. A portion of small intestine in the hernial sac gangrenous. Concave surface of the liver, here and there livid. Gall-bladder containing thick black bile, and a large calculus. A considerable extravasation of bloody serum among the viscera of the abdomen. In the thorax, the pericardium adhered firmly to the heart on all sides. The heart large and flaccid. The larynx black, livid, and gangrenous. The sinusses of the dura mater, and the vessels of the pia mater and brain, extremely turgid with frothy blood. Between the meninges, and under the pia mater, as also in the lateral ventricles, was serum. In the beginning of the spinal marrow a cavity of a large size, such as Morgagni had never before seen. The cellular membrane of the scrotum greatly inflated with air.

Remarks, Morgagni thinks the cavity above-mentioned had contained air, and remembers to have heard Valsalva say, that he had met with all the veins and the heart distended with air in a dead body. Pechlinus saw in the body of a man who had died from long-continued pain in the belly and tightness of the chest, not only the stomach and abdomen puffed up with air, but the heart immensely distended with the same. The veins of the body were also filled in many places with air, Grotzius dissected a woman who died in a miserable manner, of continual faintings, pain, and sense of suffocation, whose heart was void of blood, but prodigiously distended with air. Lastly, Ruysch found in a woman who died suddenly, a heart enlarged to an amazing magnitude with air. On thrusting the point of a knife into it, it subsided instantly, like a bladder blown up with wind. He attributes the sudden death of this fisherman to the congestion of frothy blood and bubbles of air in the brain. The various chronic or even acute lesions of the other viscera were probably owing to the impeded action of the heart from adhesion of the pericardium.

(To be continued.)

### *To the Editors of the Medico-Chirurgical Journal & Review.*

GENTLEMEN,

THE impartial conduct which the Medico-Chirurgical Journal has pursued during the course of the long agitated question relative to Yellow Fever being contagious or not, induces me to think, that it will not refuse admittance to the following document, containing a consise *exposé* of the arguments on both sides, drawn up for that great work, the French Dictionary of Medical Sciences, by two distinguished writers of a rival nation. In this, some facts which were not generally known in England will be brought forward, and upon the whole, the final decision of the French Editors on this momentous question, will, I think, be interesting to the British medical public.

I am, Gentlemen,
Yours, &c.
AMICUS.

" *Is the Yellow Fever contagious?* The history of Yellow Fever, involves an important question, which it would be most desirable to solve. —namely, whether or not it is contagious? This solution, so interesting to all nations, is particularly to be sought after by those, whose commercial communications are frequent with those countries where the fever is prevalent. Physicians of eminence, such as Chisholm, Wright, Lining, Currie, Makittrick, Pugnet, Aregula, Palloni, Cailliot, Thebaut, Bally, &c, maintain its contagious nature; while others, of not less repute, as Devèze, Valentin, Miller, Dalmas, Smith, Savaresi, &c. entertain a contrary opinion. Again, Gilbert, Clark, Humboldt, and many others have thought that the fever in question is only contagious under certain conditions of locality and temperature. Rush is well known to have first adopted, and afterwards abandoned the contagious creed. M. Moreau de St. Méry asserts, however, that Dr. Rush declared on his death-bed, that he had denied contagion for a particular reason, although he had never for a moment altered his belief in the infectious nature of yellow fever. He disavowed in his last moments every thing which he had written in favour of non-contagion. " Il a désavoué a son heure supreme, tout ce qu'il avoit ecrit en faveur de la non contagion."*

" All these authors had opportunities of seeing the disease both in America and Europe, and speak from experience. We shall now give a fair exposition of the arguments on both sides. We believe, however, that much of the discrepancy of opinion among physicians on this point has arisen from the different ideas attach-

---

* If this be true, we can attach little importance to the testimony of that physician who could thus prostitute his talents and influence at the shrine of interest. This is another specimen of American *veracity*. ED.

ed to the word *contagion*. Thus, some pathologists admit, that the matter of contagion may be transmitted to a certain distance, through the medium of the air, while the greater number consider it as resulting only from mediate or immediate contact—*contagium a contactu*. Are there not many diseases; for instance, the plague and typhus, which may be communicated in either way?

"According to Ulloa (*Relacion historica del viage a la America Meridional, &c.*) the South Sea galleons, having sailed from Panama, in 1740, for the purpose of placing the treasure in security at Guayaquil, carried to this latter colony the yellow fever, which there committed great devastation, especially among the seamen and strangers; the people of the country being nearly exempt from its influence. In 1741, some strangers introduced the disease into Malaga, by means of merchandize. (Villalba, *Epidemologia.*) Lind reports, that the trunk of a young man who died at Barbadoes of yellow fever having been opened at Philadelphia, spread the disease throughout the city. The same physician speaks of that which prevailed at Cadiz in 1764, as having been imported from America. The fever which broke out in the United States in 1793, was, according to Bally, introduced there by the French colonists who emigrated thither from the West Indies to escape massacre. The Corvette, *Dolphin*, which conveyed the governor of Havannah to Cadiz, carried to this last place the fever under consideration, which first attacked those who had communication with the baggage, but afterwards spread through the city. Great numbers of the inhabitants, who fled in all directions into the adjacent villages and country, carried the contagion along with them. Whole families who remained tn Cadiz, but kept themselves insulated, escaped the fever. (Aregula.) During the epidemic at Leghorn in 1804, we are assured, that those who avoided all communication with the sick were unaffected by the contagion. (Palloni.) In 1802, a new epidemic ravaged Philadelphia shortly after the arrival of a packet vessel from Cape François. (Bally.) Aregula observed at Malaga 1803, that on every Monday, a great number of people fell ill, from their meeting together in the churches the preceding Sunday. The yellow fever prevailed at Antequerra in 1804. After a solemn procession for imploring divine interposition, the mortality was more than doubled.

"The learned M. Thiebaut de Berneaud, who was present at Leghorn in 1804, when yellow fever desolated that city, and who addressed on this occasion, a most animated epistle to Professor Desgenettes, entertains no doubt of the disease having been imported. We shall here give some important details of M. Thiebaut, relative to this importation.

"On the 18th of August, 1804, the Spanish ship, ANNA MARIA, cast anchor in the Port of Leghorn on her return from the Havannah. During the voyage, almost the whole crew died of yellow fever. Having previously touched at Cadiz, she was refused entrance there, but what was still more inhuman, she was furnished with a fresh crew and a *bill of health !* A few days after casting anchor in Leghorn roads, two sick men quitted the vessel, and went to lodge at an inn. Three days after their debarkation they died. Twelve others, who lodged in the same house, fell in succession, and the inn itself became a focus of contagion. A Neapolitan, who lodged at this Auberge, decamped, in order to avoid the disease ; but six days after his departure, he was seized with yellow fever and died. A baker of Leghorn having sold some biscuit on board the Spanish ship, left there the bags in which it was contained, during two days, when they were brought on shore, and slept upon by the workmen of the bakehouse. These all died, as did the baker and his wife, the infection spreading through the whole house.

" M. Pachaud, of Nice, a rich merchant settled at Leghorn, purchased from his perukier a plume of feathers that had been imported in the Anna Maria from America. He was seized with the yellow fever and died, as did his wife and servant. The perukier shared the same fate.

" A French butcher, who lodged at the inn already mentioned, died of the yellow fever, as did his wife. A French officer, and the mistress of the inn, who had visited the two persons above-mentioned, during their illness, survived them only four days.

" The officers of health that were stationed on board the ship during her twelve days quarantine, almost all the caulkers that had been employed in caulking the vessel, and several inhabitants of the houses on the Mole, were stricken with yellow fever and died.

" The ship was laden with sugar, logwood, sarsaparilla, hides, &c. which goods were landed and deposited in two stores, situated in different streets. The fever committed uncommon ravages in these two streets. Two porters employed in transporting these goods died, between the fourth and seventh days : and the man who had charge of the stores, died on the second day. Such are the principal facts on which M. Thiebaut grounds his proofs of the yellow fever of Leghorn being contagious.

" *M. Moreau de St. Méry*, who lived in the midst of the most destructive epidemics of the yellow fever, for thirty years, at Martinique, St. Domingo, and Philadelphia, has often seen proofs of the yellow fever being contagious , but adds, that this dreadful character does not appertain to every epidemic of that disease.

" *M. Moreau de Jonnes*, whose testimony also is entitled to the greatest respect, is convinced that this fever is sometimes contagious; but he has seen epidemics without any infectious properties. In the memorable epidemics of 1802 and 1803, which ravaged the West Indies and several parts of the United States, the yellow fever, he maintains, was certainly contagious. Of thirty-two persons attached to the etat-major of the army in Martinique, thirty-one died, leaving M. Moreau the only survivor. At this period, the medical officers were almost all carried off by the fever. The following authentic fact, communicated to us by M.

Moreau de Jonnes himself, seems very conclusive on the question of contagion. The French Brig *Palinurus*, having, in the year 1808, moored in the harbour of Fort Royal, Martinique, was soon visited by the yellow fever. The mortality being considerable, the governor ordered her out on a cruize, in hopes of checking the disease. While at sea, she fell inwith the English brig the *Carnation*, coming from Europe, and an engagement ensued. The French captain boarded, and captured the Englishman, and the greater part of the crew of the latter were, of course, transferred to the former. Of these, a great number were seized with yellow fever. In this case, no doubt could be entertained of the disease having been communicated by contagion; since the English sailors had not set foot in any part of the West Indies, and consequently had not been exposed to the causes of yellow fever, except on board the Palinurus.

" We shall close the evidence on this side of the question, by adducing the opinion of M. Desgenettes, who has pushed his researches on the subject to the greatest length, especially as far as regards the yellow fever of Spain and Portugal. He founds his opinion of the double nature of the disease, on the observations of competent judges who have seen this fever originate and develope itself in the middle of the Peninsula, particularly in the fifteenth and sixteenth centuries, without the possibility of communication with other infected countries; and on the other hand, on the demonstrative proofs of importation and contagion, which, of late years, have multiplied so as to remove all doubt from the mind of scepticism itself. He refers to the inestimable work of Dr. Joachim de Villalba, entitled Epidemiologia Espanola, Madrid, 1803, who traces back as far as the year 476 B. C. Also to the 11th vol. of Corvisart's Journal.

" We now come to the authorities for the non-contagious character of yellow fever. M. Louis Valentin, well known by his excellent writings, and who has published a distinct treatise on yellow fever, denies contagion in every instance. This gentleman has communicated to us the following facts and reasons, on which he grounds his opinion.

" To resolve this question, it is necessary to examine it with a mind untainted by prejudice or partiality, and disciplined by long experience. It is by seeing yellow fever several times, in various places, and under all its different aspects, that we can, with any prospect of success, attempt to remove the veil of obscurity from this subject of contention among European physicians. As those who have studied and observed this fever most, are evidently entitled to the greatest degree of credit, so the medical practitioners of North and South America have a manifest advantage over all others.

" Among the physicians of the United States, there is scarcely a dissentient voice, excepting two or three of the elder members of the college of Philadelphia, particularly Drs. Currie and Hossack. Of Dr. Rush it is needless to speak. The Spanish physicians, Desessé and Mocino, who have resided a long time, the one in Mexico, the other in Peru, where they had abundant opportunity of observing yellow fever, assert, that it is entirely devoid of any contagious character. M. Humboldt also has written to this effect in the Journal Géneral de Médecine.

"If we examine the English authors on this subject, we shall find but a very small number in favour of contagion. Those English practitioners, however, who have resided longest in the West Indies, and who have had the widest field for observation in yellow fever, are not those who have *written most* on the disease. M. Valentin has conversed, or been in correspondence with many of these, and they are entirely of his opinion. We must not, he remarks, be led away by Lining or Chisholm, whose minds were evidently biassed. (" Qui ont imprimé aux esprits une mauvaise direction.") The former had but a limited experience. He has given, indeed, a very good description of the epidemic which he witnessed at Charlestown ; but he has mistaken the cause, which was local. He, as well as Rush, has led many practitioners into error. Besides, it is more than half a century since he saw the disease which he considered as imported ; but Moultrie and Mackitrick were of a very different opinion. Chisholm only saw the yellow fever in 1792, " et comme un Medecin Navigateur, s'est trop pressa d'ecrire : il s'est trompé dans presque tout ce qu'il avance." Dr. Caldwell of Philadelphia has, in the Medical and Physical Memoirs, so refuted Dr. Chisholm, that the latter has been unable to reply. Rush, Moseley, and Miller, have also shewn the sandy foundation of Dr. Chisholm's doctrine.

" During three years that M. L. Valentin resided in St. Domingo, only a few sporadic cases of yellow fever appeared in the hospitals of Cape François. But in other quarters it was prevalent, especially among the troops that were in the habit of bivouacing. At Jean Rabel, almost the whole of the soldiers were cut off by this scourge towards the close of 1791-2. and this period, says he, certainly appeared the commencement of a new era in the icterode fever, which afterwards pressed so heavily on different parts of the colony, during the horrors of the revolution ; and particularly after our flight to North America. M. Valentin is sure, that those who came from Jean Rabel to the Cape, were unaffected by fever ; and were colonists, or those long resident in the climate. It was morally impossible, therefore, that the fever could be carried by any person from Cape François to Philadelphia in 1793, when the disease manifested itself epidemically in that ill fated city. The Anglo-Americans, it is true, accused the new comers of having brought with them the contagion, although it was notorious. that no epidemic of the kind prevailed in St. Domingo at this period. The greater part of the refugees were landed tumultuously from the fleet of Vice Admiral Cambi, at Norfolk, and other parts of Virginia, without incurring any such charge. Here they became domiciliated, and here M. Valentin established an hospital of the sick soldiers and sailors; yet not an inhabitant, native or stranger, exhibited any symptoms of yellow fever this year! All the physicians who have been sent out to St. Domingo, and who have long and carefully observed the disease in question, agree in denying contagion; and are satisfied, that yellow fever is endemic, and depending on local causes.

M. Valentin has convinced himself from extensive experience, and multiplied examples, that persons affected with yellow fever, when removed from the range of febrific causes to a more elevated and healthy situation, have never communicated the disease to others:—that individuals who have nursed the sick, in these latter places, and carried their clothes, or slept in their beds, before and after death, have not contracted the fever. Can as much be said of typhus?

"Those who have opened the bodies of yellow fever subjects, have always escaped the disease, when out of the range of the effluvia that produce it. M. Valentin has seen yellow fever patients carried to the hospitals, where no fever prevailed, and there die amid numbers of people affected with common complaints, without, in any instance, communicating it to the nurses or attendants. Inoculation with the saliva, serum, or matter ejected by vomit from the stomachs of yellow fever patients—nay, the swallowing of these, have failed to propagate the disease.

"But let the inhabitants of a healthy spot remove to one where the yellow fever prevails, and consequently where the atmosphere is vitiated—let them pass a night, or even a few hours there, without entering a house where there is any sickness, and they will carry back with them, according to the degree of predisposition, the elements, the miasms, or, if you please, the germs of the disease, which will break out in the most salubrious situation a few days afterwards, but without the power of extension among those who may nurse them.

"Of the numerous examples which might be adduced of yellow fever *originating* on board ships, M. Valentin brings forward the following. The American ship Columbia, sailed from Providence during a season of health, and arrived at Marseilles in the month of August, 1802, where she performed quarantine, having previously touched at two Spanish Ports. During the voyage and quarantine, the crew had been perfectly healthy; but on their being released from quarantine, the second and third mate, four seamen, and a negro, were successively attacked with yellow fever, and all died — three of them in three separate houses of the town, the others in the Lazaret. Neither the nurses that attended, nor the surgeons that opened them, became affected with the disease; uor did any inhabitant of the town suffer from yellow fever. It was the same in 1804, when many of the Spanish, Danish, and Swedish sailors died of yellow fever, without communicating the disease to any of their attendants or physicians.. In these cases, a ship may be compared to a place on shore, where the air is stagnant, moist, warm, and tainted with various putrid effluvia. Of this, one more remarkable instance may be related. A French flotilla, carrying troops from Italy to St. Domingo, sailed from Tarentum on the 2d. of May, 1802; but being buffeted by a storm in the Mediterranean, they put into several ports, particularly Leghorn, from whence they soon proceeded on their voyage. The provisions were bad, the salt-fish was rotten, and exhaled such an abominable odour, that it was thrown overbo rd. They were forced to put into Carthagena, where eight fresh transports were hired, on board of which, the troops and provisions were embarked. The *San Nicolas de Varrios* was appointed an hospital ship, and Dr. Beguerie was placed on board in charge of the sick. The three succeeding months of June, July, and August, were excessively hot. The yellow fever broke out early in the passage, and increased in violence as the ships approached the tropic. Dr. Beguerie having himself experienced an attack of yellow fever at Madagascar, had an opportunity, on landing at St. Domingo, of seeing the same disease there. He asserts, that there was scarcely a shade of difference between the yellow fevers of the East and West, and that with which the troops were harassed on their passage from Europe.

" The history of the epidemic at New York and Philadelphia, in 1805, furnished abundant proofs of the truth of this position. About the middle of September, the atmosphere of New York was so embued with the cause of yellow fever, that in the course of two or three days after the first alarm was given, nearly fifty-thousand people deserted the city, and fled in all directions. More than ten thousand persons established a kind of camp, or temporary town, on the elevated grounds of Greenwich; to which place they removed their merchandize, furniture, public offices, &c. &c. Those who were ill with yellow fever, accompanied the others, and many who subsequently fell ill, afterwards joined them; yet in no instance was the fever communicated or spread in the new residences. It was precisely the same at Leghorn, in 1804. Not one of the refugees who fled into the country and to Pisa communicated the disease to those among whom they resided.

" Such are the arguments and facts, by which M. Valentin attempts to demonstrate the non-contagious nature of yellow fever, and which we have not exaggerated or diminished. We think it our duty here, to notice a document in the " *New Medical and Physical Journal*" for July, 1815, written by Mr. Amiel, surgeon to the forces of his Britannic Majesty at Gibraltar. In this, numerous facts, analagous to those brought forward by M. Valentin, appear equally to prove, that yellow fever is destitute of a contagious property. One of the most conclusive facts related by Mr. Amiel is this, that while the epidemic raged at Gibraltar, in 1814, the sick that were removed but a very short distance out of the town, in no instance communicated the fever to any one.

" A delicate task yet remains for us to accomplish. *Is the yellow fever contagious or not?* In the course of our researches for the materials of this article, and in our conversations with physicians and other intelligent people, who have frequently witnessed yellow fever, we have ascertained, that the general opinion among the most able and distinguished judges is, that the yellow fever is *endemic* in the greater number of places where it appears; that some epidemics are contagious, while others are not so; and, in fine, that the disease may be sometimes imported; and is capable of developing itself sporadically in those places "*qui reunissent les conditions de l' endemic.*"

The pages of the New Medical and Physical Journal, and also of the Medico-Chirurgical Journal, have all along evinced opinions so nearly similar to the above, that the Editors need not adduce their sentiments on this occasion. The document will be read with much interest by British practitioners. EDITORS.

---

*To the Editors of the Medico-Chirurgical Journal & Review.*

GENTLEMEN,

As a reader of your Journal from its first commencement in 1811, I was somewhat disappointed on seeing, in a German account of the "Recent Progress of Medicine," (Edinburgh Medical and Surgical Journal, October, 1816) an enumeration of all the British Journals, except your own. I was the more surprised at this omission, as I have recently observed your Journal quoted in the French Dictionary of Medical Sciences, under the article "Fievre;" and, as far as I recollect, it is the only periodical medical work published in Great Britain, which the Editors mention in that recondite article.

I am, Gentlemen,

A SUBSCRIBER.

Liverpool, Nov. 6, 1816.

We beg leave to inform our Liverpool friend, that we believe our Journal has not escaped the notice of the German writer. There is some confusion, and also some *error* in the passage alluded to, which our northern contemporaries *ought* to have corrected in a note. "In Great Britain, (says he) the excellent Old London Medical Journal, Duncan's Annals of Medicine, and the Edinburgh Medical and Surgical Journal were continued. *Bradley's Physical and Medical Journal also contains a rich store of observations.*" Now, the Edinburgh Editor well knew that "Duncan's Annals" and his own Journal, were *not* continued during the last ten years, which is the period embraced by the account:— On the contrary, that the former was melted down, or transmuted into the latter. He also knew that, during the period in question, there was no such thing in existence, as the "Excellent Old London Medical Journal," unless the "London Medical and Physical Journal" be meant' which we believe to be the case.—

These points adjusted, "Bradley's Physical and Medical Journal," means the "New Medical and Physical Journal," first conducted by Dr. Bradley, and subsequently by the present Editors.

But, however flattered the MEDICO CHIRURGICAL JOURNAL may be by the notice of illustrious foreigners, it is far from considering this as any certain criterion of merit. Till very lately, what may be termed the *policy* of the Journal, was entirely neglected, and it was cast destitute and friendless on the world, to find its way as it could! Still it rose, both in credit and circulation. The eyes of the Editors, however, are opened; and their Journal will henceforth assert its rank, and make itself known wherever the English Language prevails. EDITORS.

---

### *To the Editors of the Medico-Chirurgical Journal & Review.*

GENTLEMEN,

The old adage, that "There is nothing new under the Sun," was forcibly brought to my recollection the other day, when perusing Dr. Balfour's Method of treating Rheumatism by Flagellation. I can have no wish to detract from the merits of this discovery; for I believe it to be *original* as far as respects the gentleman in question; but I can assure him, and so can hundreds who have visited China, that the practice there is very common, and most probably has been so time immemorial. The plan among these people is, to beat the part gently with thin pieces of wood till a redness is produced, when it is bound pretty tightly up in calico; and this operation is repeated every day till the pain gives way. This remedy is not confined to chronic rheumatism alone, but is applied to pains of all kinds, unaccompanied by inflammation. It is said to be very successful.

And now that the pen is in my hand, I shall mention a curious mode of treatment in two other diseases, which I have often seen practised in Bengal, and other parts of the East. The first time that I went up the Ganges, in a large budgerow, I was, one day, startled by a very unusual bustle over my head. On running up to see what was the matter, I found all the men belonging to the budgerow piled one over another on a poor fellow beneath, who appeared to be suffocating! Imagining that they were murdering the man, I commenced a war of kicks and blows on the upper strata, and dislodged them in a trice; but I was soon undeceived, by being informed, that they were curing one of their comrades of an ague fit. The plan was, whenever the first shiver was perceived, to swallow a large dose of chillies, and then lying down on a mat, every man in the vessel threw himself on the patient, with his face downwards. The patient thus lay inhaling the breaths and receiving the heat from his numerous Doctors, till perspiration burst from every pore, which usually took place in a quarter of an hour, when he was released from his confinement, and the fit was terminated. It must be kept in mind, that these gentry go entirely naked, except a small cloth between the thighs, in the manner of a fig-leaf. Nothing, therefore, interposed between the bodies of the patient and his friends; and, consequently, the transmission of animal heat was uninterrupted by any nonconducting vestments. Rough as is this treatment, I question whether the College of Physicians could devise a more speedy or effectual check to the tertian fiend.

Cholic is a complaint to which the boat-men and others employed upon the Ganges, are very subject; and appears, in most instances, to be spasmodic—probably arising from their vegetable food, and frequent exposure to wet It is also cured by manual or mechanical means. The person suffering under cholic, lies down on a mat, when two of his companions, having greased his belly well with ghee, begin to knead and pound his guts with their clenched fists till the cholic is removed, which is generally made known by some *loud reports* of artillery, the usual signal that the enemy has fled.

I am, Gentlemen,
Yours, &c.
ORIENTALIS.

---

*To the Editors of the Medico-Chirurgical Journal & Review.*

GENTLEMEN,

As I perceive that you are by no means *au fait* at the management of a table of contents, I hereby transmit you a specimen of what may be done by a little management in this way.

On opening one of the London Medical Journals for September, 1816, I was gratified by the sight of a most splendid bill of fare; and having two of my own children very ill with hooping-cough, the following title of an article in the *second page* of "contents" immediately arrested my attention. "*Dr. T. Gumprecht, on the Use of Lactuca Virosa in Hooping-Cough.*" I eagerly turned to the 235th page of the Journal, when, mournful to relate! the following was the *whole* of the article:—"*On the Use of the Lactuca Virosa in Hooping-Cough, by T Gumprecht, M. D.*"!

I am, Gentlemen,
SAT VERBUM SAPIENTIBUS.

Bath, November 10, 1816.

---

*The following are the Outlines of a Case with which we were favoured by* MR. STEVENSON, *Great Russell Street, Bloomsbury Square.*—A young Lady, seven years of age, was affected with severe pains, resembling those of Tic Douloureux, in the palpebra and other appendages of the left eye, recurring at uncertain intervals of a few hours, and lasting for several minutes. A blister was applied to the temple, and behind the ear of the affected side, and some local remedies employed, together with gentle laxatives by an apothecary, without effect, when the patient came under the care of Mr. S. who immediately ordered an emetic and brisk cathartic to be exhibited. The pain never recurred after the first of these medicines had done its office; and the second, by its operation, evacuated a large coral bead, which the child *then* recollected to have swallowed, how long before the accession of the symptoms is not stated. It is to the presence of this substance in the primæ viæ Mr. S. attributes the origin of this complaint; the removal of which, consequently, terminated the disease.—This is one of those cases sometimes happening to lucky practitioners, in which the accidental discovery of an obscure cause or circumstance of a disease impresses the bye-standers with wonder at the sagacity of the physician, and obtains for him a reputation little inferior to that of a conjuror among the vulgar, for his knowledge of the deposits of stolen goods. It is probable, the coral bead had little to do in producing the symptoms; for were such a cause adequate to this effect, we should scarcely see so many hundred persons swallowing extraneous bodies with impunity, as the stones of fruit for instance, which are not known to be otherwise injurious than by occasionally forming a nucleus for the accumulation of mucus; and at length, by their bulk, producing mechanical obstruction of the alimentary canal. We have, within these few months, cured many persons of violent painful affections of the face, resembling *tic douloureux;* some of which, indeed, had been so called by physicians of eminence, by similar means to those employed by Mr. S. There were such evident symptoms of gastric derangement, as to induce us to exhibit an emetic and purgative for the alleviation or removal of these, previous to employing remedies appropriate to the nervous affection; and in executing this first intention, the whole fabric of the disease has, as it were, fallen, and left the patient in a state of perfect recovery.

---

*Dr. Rindler's (of Midtersill in the Dukedom of Salzburgh) new method of curing cloven-footed children with strips of adhesive plaster in lieu of the machine.*—A woman, from Newkincken, brought her ninth child, 6 months old, to Dr. R. for his advice and assistance; who, on examination found it to be a well formed boy in every respect excepting the left foot, which was cloven, and so much turned inwards, that the toes were only an inch and a half distant from the heel; the child stood entirely on its exterior margin and external condyle, the remaining part of the foot being quite natural. There was a scar on the condylus externus, the consequence of splints and hard bandages, which a surgeon, under whose care the child was formerly placed, had unsuccessfully employed. Mr. Weidinger, under Dr. R's directions, undertook to treat the child agreeably to the new method, and at the third application of the strips, they had the satisfaction to see the approaching success of the treatment. This method was regularly and carefully repeated daily, and in the fourth week, the child stood almost as well on the sole of that foot, as on the other. The mother, on account of her family, being desirous of returning home, was dismissed, having previously been instructed in the mode of applying the plaster strips, and Mr. W. occasionally visited them. In the eighteenth week of the treatment, the form of the foot, and the flexion of the knee and foot-joint, were quite perfect, and the child was completely cured.

About the same time, a boy, 6 months old, was brought to Dr. Rindler on account of his left thumb, which had been bent inwards from his birth, and which was found to be occasioned solely by the muscles and ligaments. The adductor was rubbed with warm oil, and its antagonist with spirits of wine, in order to relax the one, while the other was strengthened. The hand was then well wiped, and strips of linen spread with the empl. oxycroceum were applied in such a manner, that the first strip ran from the back of the second phalanx, over its interior margin, outwards, in the direction of the extensor, *tour à tour;* the second strip on the first phalanx, in the same direction, and on the head of the metacarpal bone of the thumb, in the direction of the adductor. By these means the boy was entirely cured in three weeks. Encouraged by the success of this method, Dr. R. intends to try it in several other kinds of deformities, even where the bone itself is in a faulty state, as crooked knees, legs, necks, lateral curvatures of the spine, rachitis, and particularly in infantile umbilical hernia.

Dr. Villermi, in treating of the formation of pseudo-membranes, divides the period of their formation into four stages. In the first, the previously natural membrane, from which the pseudo-membrane takes its rise, shews a greater number of vessels on its surface. Small, distinct, and coherent points, are seen in the shape of a kind of medullary flakes, generally of a whitish colour, appearing like gauze, which are very short, and readily disappear when rubbed. The second stage begins as soon as the exhaled matter assumes a membranous appearance, and is characterized by the increased thickness of the pseudo-membrane. The third stage commences at the time when the vessels make their appearance in the new-formed membrane; till that time it appears like coagulated lymph, assuming afterwards a fatty appearance. In the fourth stage, about the 21st day, vessels are seen in the new-formed membrane; these are continuations of the vessels of the original membrane. The pseudo-membrane, which hitherto consisted chiefly of albumen, in this period is changed into real cellulosa; losing its fatty appearance, and becoming thinner, it presents thin, transparent, soft serous lamellæ, as they are contained in the cellulosa. The pseudo-membrane now becomes itself an exhaling organ, like other serous membranes, and is capable of becoming inflamed by irritating causes; and thus, another pseudo-membrane can be formed on the top of the first. They are met with on the arachnoidea, peritonæum, pericardium, tunica vaginalis of the testicles, but chiefly on the pleura. In women of the town, adhesions have been found frequently between the ovaria and the peritonæum.

Dr. V. puts the question, whether that reddish jelly, frequently found in the joints about the articular membranes, may not be considered as a produce of inflammation.

The pseudo-membranes, like the serous membranes, never contain any fat. If they are lacerated by any new serous inflammation, they form a kind of process; which, under the microscope, has the appearance of the processus epiploon.

In the membranes produced in the croup, small longish fibres and vascular ramifications are said to have been noticed.

Dr. MERRIMAN and Dr. LEY will begin their next Course of Lectures on Midwifery and the Diseases of Women and Children, at the Middlesex Hospital, on Saturday, December 14, at half past ten o'clock.

DIED. — At Cheshunt, in Hertfordshire, in consequence of a fall from his horse, Thomas Sanders, M. D. in the 70th year of his age. — At his house in Mark Lane, aged 78, J. H. Sequeira, M. D.—At Berlin, in the 70th year of his age, Dr. Bremer, who greatly distinguished himself in promoting Vaccination.

