# Errata

**Published:** 1816-07

**Authors:** 


					528, line 10. For lution, read solution.
? ? v J ,? M J ? iiii

				

